# An acquired factor V inhibitor induced uncontrolled bleeding in a postsurgery patient

**DOI:** 10.1002/ccr3.3452

**Published:** 2020-12-01

**Authors:** Riccardo Bruna, Riccardo Moia, Alessandra Valpreda, Enrico Dosio, Roberta Rolla, Augusto Federici, Umberto Dianzani, Gianluca Gaidano, Andrea Patriarca

**Affiliations:** ^1^ Division of Hematology Department of Translational Medicine Università del Piemonte Orientale Novara Italy; ^2^ Regional Center for Hemorrhagic and Thrombotic Diseases Haematology Laboratory City of Health and Science University Hospital of Molinette Turin Italy; ^3^ Department of Health Sciences and Interdisciplinary Research Center of Autoimmune Diseases (IRCAD) Università del Piemonte Orientale Novara Italy; ^4^ Department of Oncology and Oncohematology Hematology and Transfusion Medicine L. Sacco University Hospital University of Milan Milano Italy

**Keywords:** acquired hemophilia, bypassing agents, factor V inhibitor

## Abstract

This case report highlights the challenges in controlling bleeding and correcting coagulation tests through the use of bypassing agents in patients with FV inhibitors.

## Graphical abstract

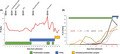

## 1 CASE PRESENTATION

We report a case of a 90‐year‐old man who developed a high titer (140 Bethesda Units) FV inhibitor that impairs the activity of all coagulation factors except FVIII. The patient was treated with bypassing agents, including nonactivated and activated prothrombin complexes, and recombinant FVIIa, as well as with immunosuppressive therapy. Despite a significant reduction in the antibody titer and the despite the multiple mechanisms of bypassing agents administered, the coagulation parameters did not significantly improve and the patient developed a fatal bleeding.

A 90‐year‐old man was referred to our institution for hematuria and abnormal coagulation tests. The patient had an unremarkable clinical history till 1 month before admission to the Surgery department, when he underwent a cholecystectomy for gangrenous cholecystitis. Surgery was successful without significant complications, and the patient was discharged from the Surgery department on prophylactic antibiotic therapy with amoxicillin‐clavulanic acid. At the time of surgery, coagulation tests were normal and the patient did not show unexpected perioperative bleeding.

After 2 weeks, the patient presented to the emergency department for persistent hematuria. Blood tests showed severe normochromic normocytic anemia (Hb 8.6 g/dL), a platelet count of 271.000/µL, and altered coagulation tests, with a PT‐ratio of 8.36 and an activated partial thromboplastin time (aPTT) >160 seconds (aPTT ratio nonevaluable). The patient was not taking any medication that may interact with coagulation. A challenge with fresh frozen plasma and vitamin K administration failed to improve the coagulation tests. The results of the abnormal coagulation parameters did not change after mix test with healthy donor plasma, consistent with the presence of an antibody pan‐inhibiting multiple coagulation factors.

At admission in the Haematology department, the patient was asymptomatic except for hematuria. Physical examination was unremarkable with only a slight reddening of the skin around the cholecystectomy scar. Cystoscopy and PET/CT scan did not reveal sites of detectable neoplasia. Coagulation tests confirmed the prolongation of the PT‐ratio and aPTT ratio; other laboratory parameters are detailed in Table 1. The activity of all tested coagulation factors (FII, FV, FVII, FIX, FX, FXI, and FXII) was <5%, with the exception of FVIII (62%). In addition, thrombin time was normal and the thromboelastogram revealed abnormal platelet aggregation and coagulation.

**TABLE 1:** Laboratory data

Variable	Reference range	On admission
White cell count/μL	4500‐11000	9890
Differential count/μL		
Neutrophils	1800‐7700	6920
Lymphocytes	1000‐4500	1800
Monocytes	0‐800	1050
Eosinophils	0‐450	70
Basophils	0‐200	50
Hemoglobin (g/dL)	13.5‐17.5	8.6
MCV (fL)	80.5‐99.7	86.4
MCH (pg)	26.6‐33.8	27.9
Platelet count/μL	150 000‐450 000	271 000
PT‐ratio	0.8‐1.2	8.36
aPTT ratio	0.8‐1.2	Not evaluable
Fibrinogen (mg/dL)	180‐400	491
Factor II		<5%
Factor V		<5%
Factor VII		<5%
Factor VIII		62%
Factor IX		<5%
Factor X		<5%
Factor XI		<5%
Factor XII		<5%
Creatinin (mg/dL)	0.9‐1.3	0.97
Uric acid (mg/dL)	3.5‐7.2	5.2
Sodium (mEq/L)	134‐146	135
Potassium (mEq/L)	3.5‐5.5	4.1
Total bilirubin (mg/dL)	0.3‐1.2	0.3
LDH (U/L)	208‐450	317

Abbreviations: aPTT, activated partial thromboplastin time; LDH, lactate dehydrogenase; MCV, mean corpuscolar volume; MHC, mean corpuscolar hemoglobin; PT‐ratio, prothrombin time ratio.

Laboratory tests performed in our patient presenting with clinical bleeding suggest the presence of an acquired inhibitor against a coagulation factor that is active at the convergence of the two coagulation pathways and that may also have a role in platelet aggregation. On these grounds, a FV inhibitor should be considered as a main culprit for this clinical scenario according to some previous reports.^1^ After performing plasma dilutions, all coagulation factors, except FV, returned to normal levels at dilution 1:5, 1:10. More precisely, at 1:80 dilution, the FV activity was still <10% demonstrating that the inhibitor was specific to FV. Consistently, a FV inhibitor was detected with a 134 Bethesda Unit (BU) titer.

In order to reduce the uncontrolled bleeding and the production of the FV inhibitor, therapy with prothrombin complex and steroids was started. On day 5 of treatment, only a slight improvement in the PT‐ratio (up to values between 5.5 and 6), but not in aPTT ratio, was achieved. Fourteen days after the start of treatment, a second inhibitor titer was performed, showing the persistence of the antibody at high titer (134 BU). Consistently, immunosuppressive therapy was modified adding low dose cyclophosphamide (100 mg/die orally) and rituximab (375 mg/m^2^, planned 4 weekly doses).

**FIGURE 1:**
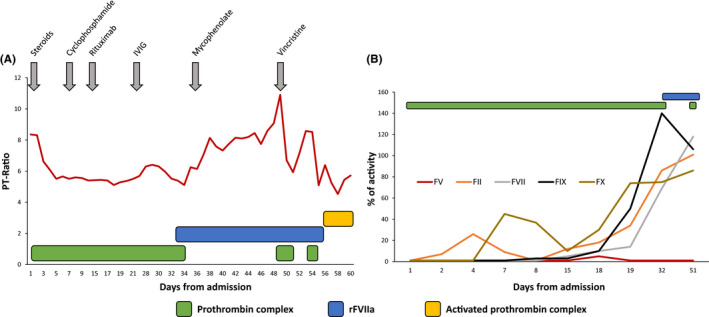
Timeline of the patient's PT‐ratio and coagulation factors activity. A, The red line denotes the timeline of the patient's PT‐ratio during the clinical course in relationship with therapy. Prothrombin complexes are represented by the green boxes, rFVIIa by the blue box, and activated prothrombin complex by the yellow box. The sequence of the various attempts of immunosuppressive therapies is indicated by gray arrows. B, The activity of coagulation factors (FV, red; FII, orange; FVII, gray; FIX, black; and FX, brown) is represented over time in relationship with administration of the prothrombin complex (green boxes) and rFVIIa (blue box) administration. The activity of coagulation factors was tested from admission until day 51

After 30 days of immunosuppressive therapy and daily administration of prothrombin complex, no improvement was evident and immunosuppressive therapy was modified by adding mycophenolate (2 g/day) and vincristine (2 mg) instead of cyclophosphamide. Moreover, bypassing therapy was switched from prothrombin complex to recombinant activated factor VII (rFVIIa). However, after few days of rFVIIa, the patient's coagulation tests worsened (Figure 1), as well as hematuria. Therefore, rFVIIa was initially administrated together with prothrombin complex with improvement in both clinical and laboratory response. However, rFVIIa and prothrombin complex did not completely resolve hematuria and were replaced with activated prothrombin complex. The trend of PT‐ratio and of coagulation factors and their relationship with treatment are reported in Figure 1. The aPTT ratio was always elevated, irrespective of the immunosuppressive and bypassing treatments administered.

After more than 2 months of immunosuppressive therapy, the FV inhibitor titer reduced from 140 BU to 5 BU. However, despite several types of bypassing products (ie, prothrombin complex, rFVIIa, and activated prothrombin complex) complemented by supportive therapy requiring red blood cells transfusions every 2‐3 days due to persistent hematuria, the patient died for an acute bleeding event.

## 2 DISCUSSION

The development of FV inhibitors can occur at any age may display a variety of clinical symptoms, ranging from asymptomatic laboratory abnormalities to life‐threatening bleeding events.^1^ The majority of FV inhibitors described in the past decades were autoantibodies arising after the exposure to topical fibrin glues or bovine thrombin preparations that were contaminated with bovine FV.^2^, ^3^ Currently, these glues are no longer used, and in fact, they were not utilized at the time of surgery in our patient. In a systematic review evaluating published cases of acquired FV inhibitors, the association with predisposing factors could be summarized, with some overlapping cases, as follows: (a) recent exposure to antibiotics (42%); (b) recent surgical procedure (31%); (c) recent infection (23%); (d) cancer (22%); and (e) an underlying autoimmune disorder (13%).^1^ Our patient displayed at least three of the abovementioned risk factors, namely antibiotic exposure, recent infection (ie, gangrenous cholecystitis), and surgical procedure, while no cancer or autoimmune disorders were clearly identified.

Therapy of FV inhibitors is a therapeutic challenge and should be focused on two different objectives: (a) restoring coagulation in order to treat the bleeding symptoms; and (b) trying to eradicate anti‐FV antibodies. Prothrombin complex or rFVIIa are generally used to recover from acute bleeding events.^1^ In our case, prothrombin complex and activated prothrombin complex seemed to have efficacy in limiting bleeding, whereas rFVIIa was less efficacious. Since the mechanism of these bypassing agents is different, it may be possible that the nature of the anti‐FV antibody influences their therapeutic activity in the individual patient. Platelet transfusions, which should provide FV protected from the possible inhibition of the antibody, have demonstrated conflicting results.^4^ However, the combination of prothrombin complex with platelet transfusions may enhance the activity of prothrombin complex.^5^

Interestingly, a systematic review of FV inhibitors has showed that the inhibitor titer does not correlate with the level of FV deficiency nor with the bleeding risk, whereas a positive correlation exists between residual FV activity and bleeding symptoms.^6^ In accordance with these data, also in our case report the immunosuppressive therapy allowed the achievement of a progressive reduction in the inhibitor titer, although FV levels always remained <1% leading to persistent bleeding. This case report also highlights the challenges in controlling bleeding and correcting coagulation tests through the use of bypassing agents, since bleeding as well as PT‐ratio and aPTT were never fully controlled despite the use of multiple bypassing agents with distinct mode of actions. Rare acquired coagulation disorders are still an unmet clinical need with the current therapeutic strategies, and novel therapies are needed.

## CONFLICTS OF INTEREST

Gianluca Gaidano has to disclose roles in advisory boards or speakers' bureaus of the following companies: Astra‐Zeneca, Sunesys, Abbvie, and Janssen. All the other authors have nothing to disclose.

## AUTHOR CONTRIBUTIONS

All the authors included in this work contributed to patient care and with manuscript preparation.
